# Switching Power Universality in Unipolar Resistive Switching Memories

**DOI:** 10.1038/srep23930

**Published:** 2016-04-01

**Authors:** Jongmin Kim, Kyooho Jung, Yongmin Kim, Yongcheol Jo, Sangeun Cho, Hyeonseok Woo, Seongwoo Lee, A. I. Inamdar, Jinpyo Hong, Jeon-Kook Lee, Hyungsang Kim, Hyunsik Im

**Affiliations:** 1 Division of Physics and Semiconductor Science, Dongguk University, Seoul 100-715, Republic of Korea; 2 R&D Division, Flash Integration Technology Team, SK-Hynix, 2091 Gyeongchung daero Bubal-eub, Icheon-si Gyeonggi-do, Republic of Korea; 3 LED Product & Technology, LED Business, Samsung Electronics Co., Ltd, San #24 Nongseo-dong, Giheung-gu, Yongin-city, Gyeonggi-do, 446-711, Republic of Korea; 4 Department of Physics, Hanyang University, Seoul 133-791, Republic of Korea; 5 Center for Opto-Electronic Materials and Devices, Korea Institute Science & Technology (KIST), Seoul 136-791, Republic of Korea

## Abstract

We investigate the resistive switching power from unipolar resistive switching current-voltage characteristics in various binary metal oxide films sandwiched by different metal electrodes, and find a universal feature (the so-called universality) in the switching power among these devices. To experimentally derive the switching power universality, systematic measurements of the switching voltage and current are performed, and neither of these correlate with one another. As the switching resistance (*R*) increases, the switching power (*P*) decreases following a power law *P* ∝ *R*^−β^, regardless of the device configurations. The observed switching power universality is indicative of the existence of a commonly applicable switching mechanism. The origin of the power universality is discussed based on a metallic filament model and thermo-chemical reaction.

Though reversible resistive switching (RS) is not novel: it has been observed in various insulating oxides sandwiched by metal electrodes since the 1970s^1^,^2^,^3^,^4^,^5^,^6^,^7^,^8^,^9^,^10^,^11^, there is renewed interest in using the RS phenomena as an operating principle for new-functional nonvolatile memory (often referred to as ReRAM) applications. According to the way reversible RS phenomena are controlled, either by current magnitude or by voltage bias polarity, the character of RS is grouped into two categories: unipolar or bipolar. The responsible physics of the RS phenomena is diverse. Many models proposed include oxygen diffusion^12^, Schottky barrier behavior at the metallic contact interface^13^, charge trapping/de-trapping^14^, and the creation of crystalline defects^15^.

The generally accepted RS model in binary metal oxide films exhibiting unipolar RS is a filament model such that a conduction channel called a filament is formed in the insulating oxide film if a critical voltage is applied across the film. Although the filament model is widely accepted in binary metal oxide based unipolar ReRAM^16^,^17^,^18^, the common underlying physics of the model is not well clarified regardless of the materials used. So, the absence of quantified common features (in another word, universality) in the RS phenomena makes it difficult to accept the general applicability of the filament model. From a practical point of view, predictable universal device parameters are very useful for device and circuit engineers to understand the correlation between material, device, and circuit at the industry level (see Supplementary Information, Fig. S3) and eventually as a starting point of their advanced research^19^. Recently, scaling effects in RS devices have been reported^20^,^21^,^22^,^23^,^24^. However, discovery of additional universal behaviors in which two main parameters, switching voltage and current, are involved would certainly be useful in gaining an intuitive understanding of switching mechanisms and practical RS memory applications.

In this work, we present the RS characteristics of various binary oxides sandwiched by metal electrodes and report on the existence of universality between the switching power (*P*) and switching resistance regardless of the oxide materials and metal electrodes used. In order to validate the observed universality of resistive switching powers in binary metal oxide based unipolar ReRAM devices, as many binary metal oxides as possible: NiO, TiO_2_, Nb_2_O_5_, CaO, MgO, HfO, MnO_2_ and Al_2_O_3_ which show clear unipolar RS characteristics were investigated. We describe the power universality and low-resistance current-voltage (*I*–*V)* features using a general electro-thermal chemical reaction model and the filamentary resistive switching model.

## Results and Discussion

**Figure 1:**
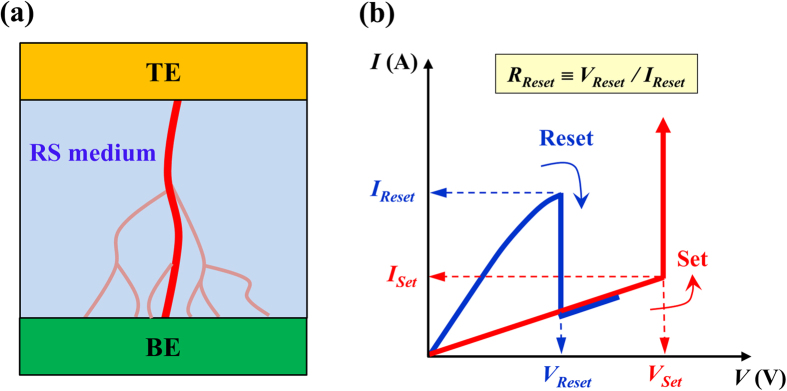
Schematic diagram for filamentary channels in a RS device and unipolar RS characteristics. (**a**) Schematic of an electrode-binary oxide-electrode RS device with a tree structure of filamentary conducting channels embedded inside the RS insulating medium. (**b**) Typical unipolar-type resistive switching *I*–*V* characteristics of a metal-binary oxide-metal RS device. The switching resistance *R* is defined as the ratio of the switching voltage to the switching current.

The RS memory device consisted of a highly resistive binary oxide film which is sandwiched by a top electrode (TE) and a bottom electrode (BE), as illustrated in Fig. 1(a). When a large voltage is applied to the pristine metal-dielectric insulator-metal RS device, a process called “forming” (which changes the insulating high-resistance phase into a bistable reversible switching phase between the high-resistance state (HRS) and the low-resistance state (LRS)) occurs (see Fig. 1(b)). Afterwards, by sweeping the bias voltage, an abrupt drop in the current appears at a relatively lower voltage (named the Reset voltage). Then, by re-sweeping the voltage, a similar abrupt increase in the current occurs at a higher voltage (named the Set voltage).

**Figure 2:**
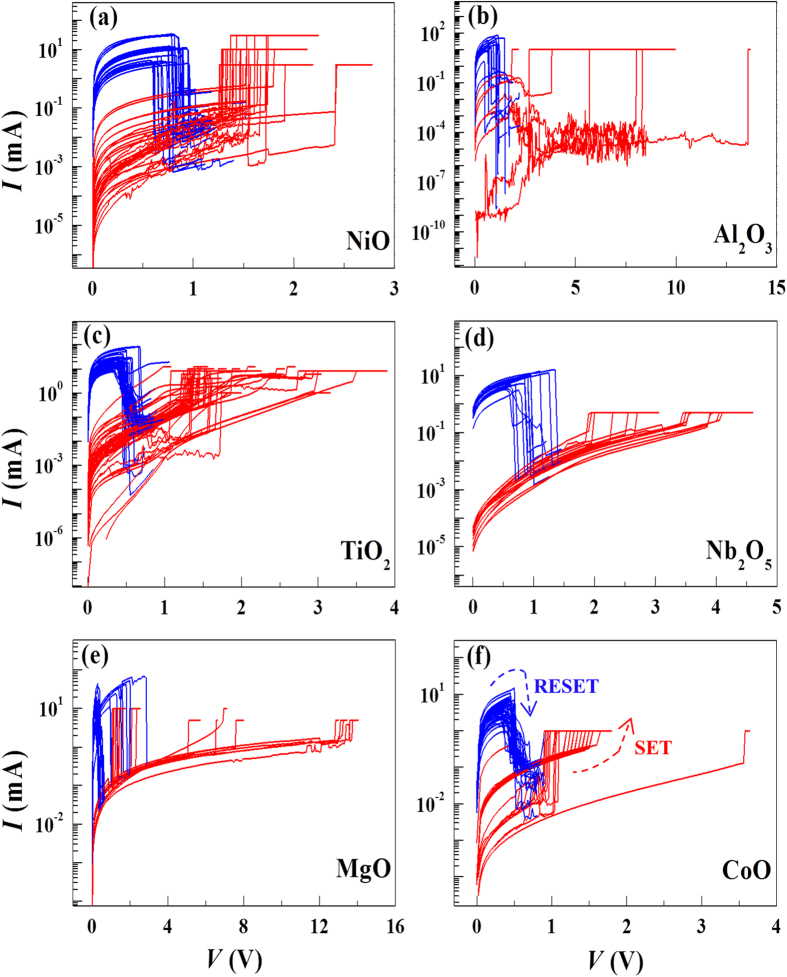
Resistive switching *I*–*V* characteristics for various metal-oxide-metal systems. (**a**) NiO, (**b**) Al_2_O_3_, (**c**) TiO_2_, (**d**) Nb_2_O_5_, (**e**) MgO, (**f**) CoO. The blue and red curves show the LRS and HRS *I*–*V* characteristics respectively. The blue and red arrows in (**f**) represent the Reset (LRS→HRS) and Set (HRS→LRS) processes, respectively.

Figure 2 shows the bistable RS current-voltage (*I*–*V*) characteristics for various metal-oxide-metal ReRAM devices (See Supplementary Information, Fig. S1). For all devices, the electrical forming process occurred with a wide range of 3 V and 20 V. The observed switching *I*–*V* characteristics are typical of unipolar-type RS behavior. The temperature (*T*) dependence of the transport channel in the LRS is very similar to the *T*-dependent electrical conduction in metals, suggesting that the physical object responsible for the LRS transport is metallic^25^. The observed unipolar switching behavior in our binary oxide devices is well described by the metallic filament model^26^,^27^,^28^. The physical formation of the metallic filament in binary oxides has been directly identified by ourselves^19^,^29^,^30^ and other groups^31^,^32^.

**Figure 3:**
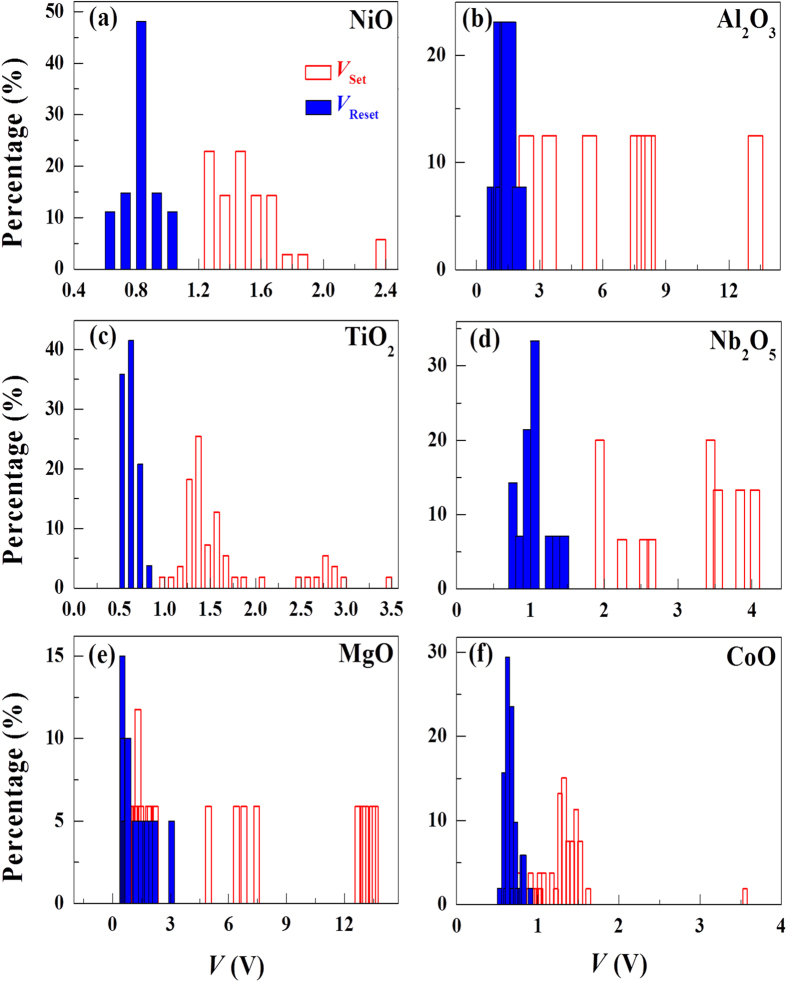
Distributions of switching voltages. There seems to be no obvious common features in the switching voltage among devices. (**a**) NiO, (**b**) Al_2_O_3_, (**c**) TiO_2_, (**d**) Nb_2_O_5_, (**e**) MgO, (**f**) CoO.

**Figure 4:**
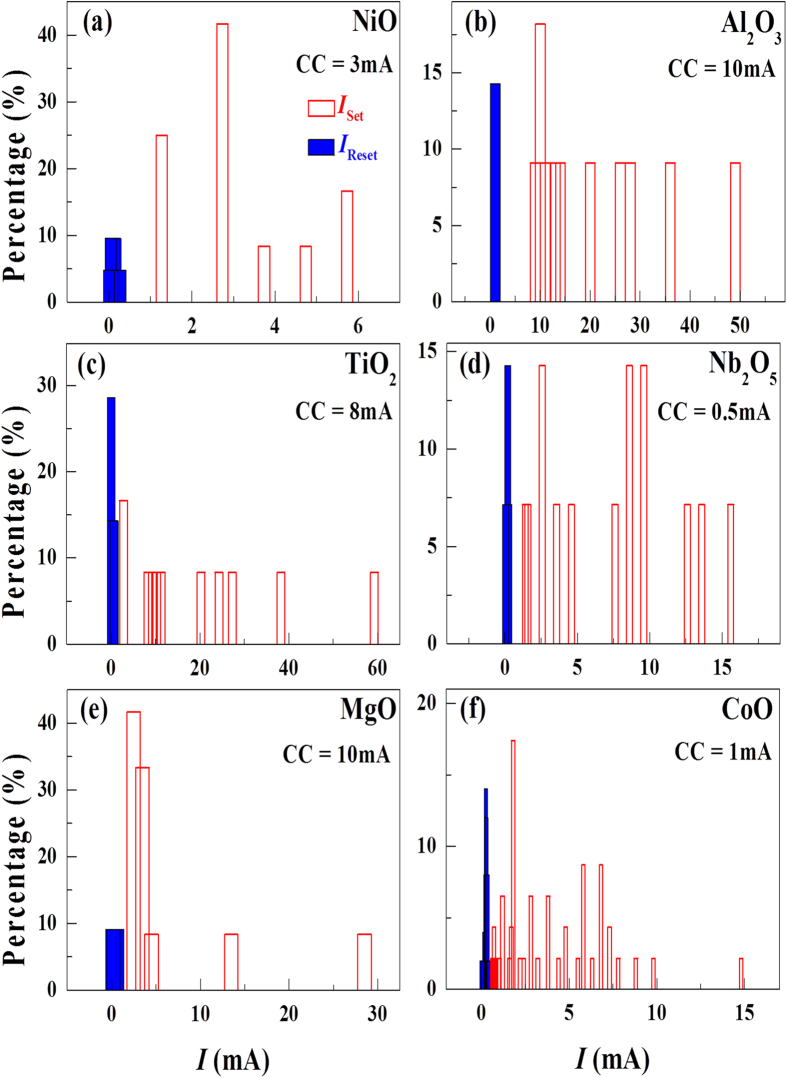
Distributions of switching currents. There seems to be no obvious common features in the switching voltage among devices. (**a**) NiO, (**b**) Al_2_O_3_, (**c**) TiO_2_, (**d**) Nb_2_O_5_, (**e**) MgO, (**f**) CoO.

**Figure 5:**
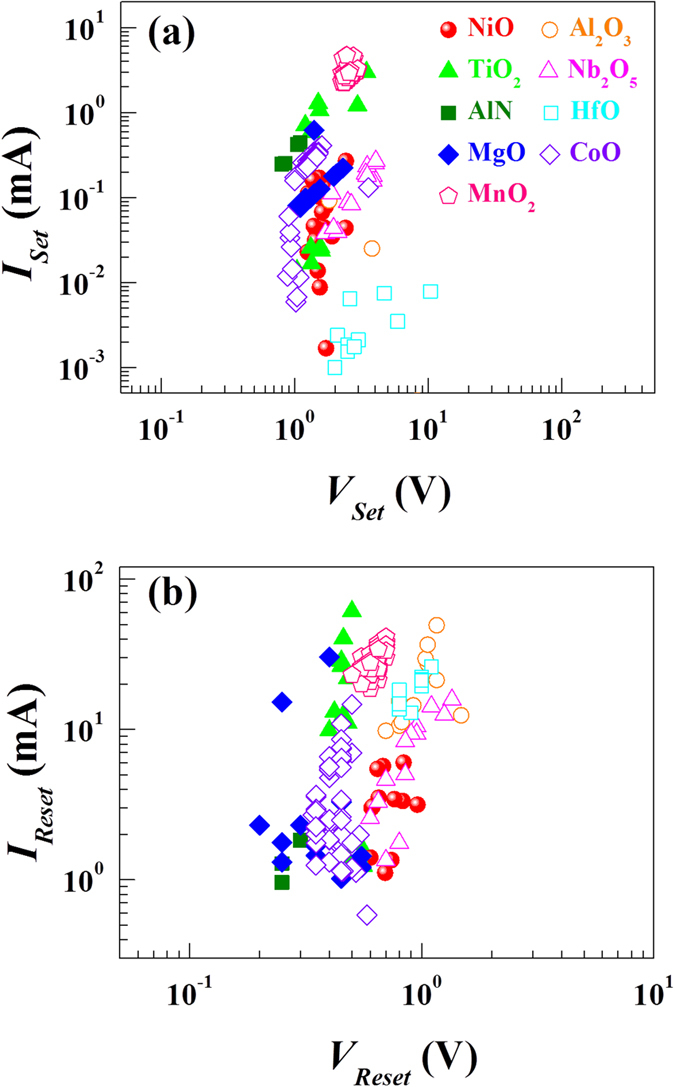
Non-correlation between switching voltage and switching current. Scatter plot of switching voltage versus switching current for (**a**) the Set (HRS→LRS) and (**b**) the Reset (LRS→HRS) processes. There seems to be no correlation between them.

Figure 3 shows the measured switching voltages for the LRS→HRS (Reset) and HRS→LRS (Set) processes. The distribution in the switching voltage appears to be random without noticeable common trends among the devices. Similarly, the measured switching currents of each device are randomly distributed and there does not seem to be a common trend in the distribution of the currents (see Fig. 4). Furthermore, the measured switching voltage and current values do not correlate with one another, as clearly evident in the scatter plot of Fig. 5.

The mechanism responsible for the LRS→HRS switching can be due to either redox-oxidation or melting (rupture) of the main filament. However, considering that the effective temperature of a metallic nano-wire for redox-oxidation (a few hundred °C) is much lower than that for melting (a few thousand °C), a thermo-chemical redox-oxidation process is more likely to be responsible for the LRS→HRS switching^33^. At a current large enough to initiate the thermal chemical reaction between the metallic element constituting the filament and un-bonded oxygen nearby, redox-oxidation starts and breaks the filament causing an abrupt drop in current (LRS→HRS switching)^34^,^35^. In each resistive switching cycle, a different formation of metallic (filamentary) channel structures is anticipated resulting in the fluctuation of switching voltage and current. In addition, unbroken high-resistive filaments can still remain after the Reset process contributing to the HRS current^25^.

**Figure 6:**
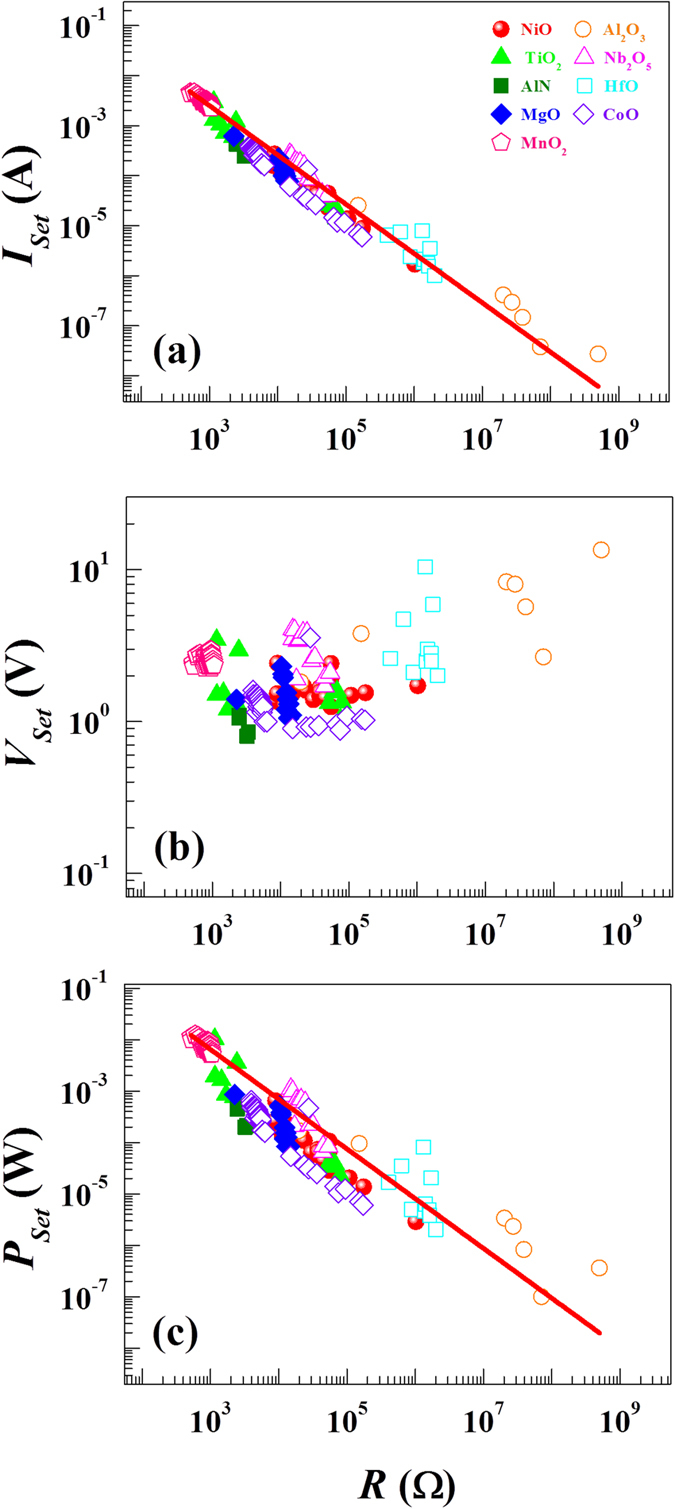
Switching current and switching power universality for the Set process. (**a**) Switching current as a function of *R*. (**b**) Switching voltage as a function of *R*. (**c**) Switching power as a function of *R*. While the switching current and power show a universal behavior which can be described by a power law, there seems to be no universal feature between the switching voltage and the switching resistance. The solid lines represent the fitting curves based on a power law relationship.

**Figure 7:**
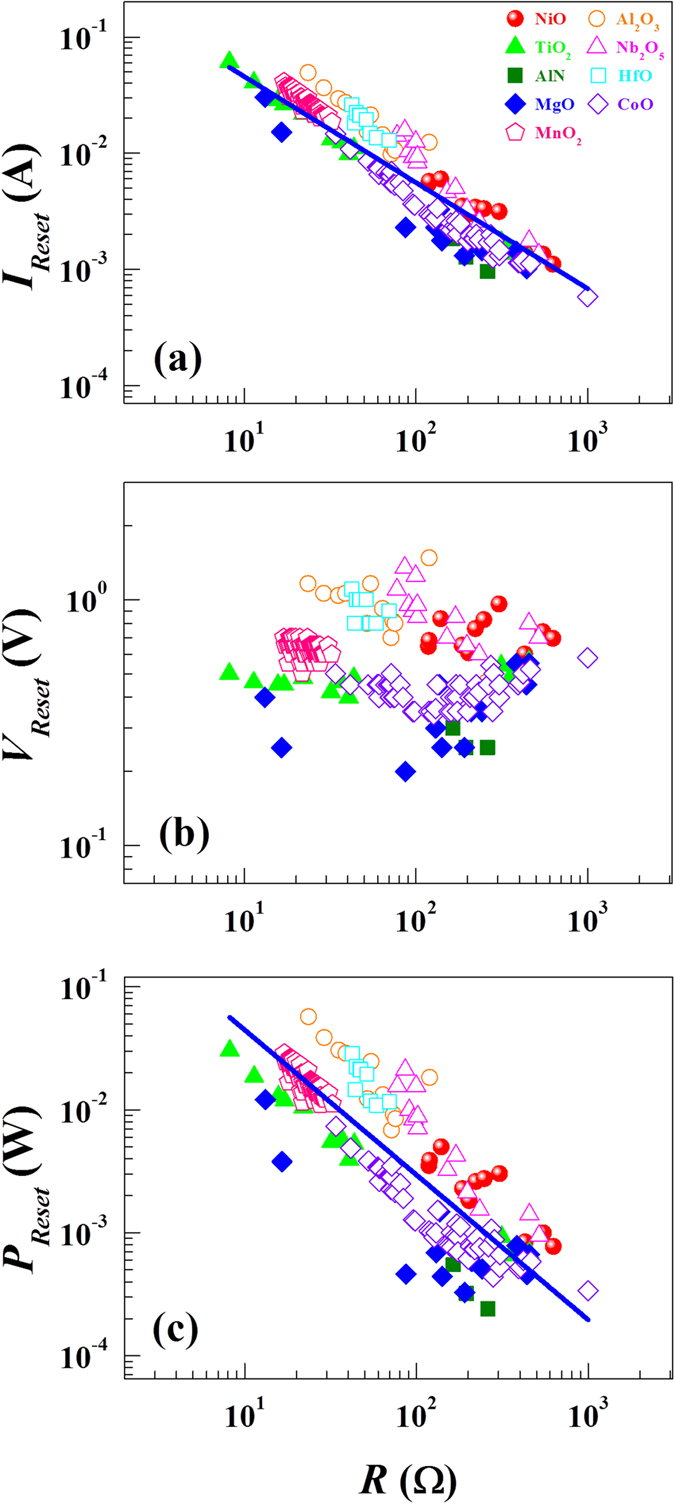
Switching current and switching power universality for the Reset process. (**a**) Switching current as a function of *R*. (**b**) Switching voltage as a function of *R*. (**c**) Switching power as a function of *R*. While the switching current and power show a universal behavior which can be described by a power law, there seems to be no universal feature between the switching voltage and the switching resistance. The solid lines represent the fitting curves based on a power law relationship.

Scaling effects between switching current and switching resistance have been demonstrated for the Reset process^21^,^22^. In order to confirm the existence of such scaling effects in our RS devices, we plot both the switching current and the switching voltage as a function of switching resistance for the Set and Reset processes. The switching resistance *R* is defined as a ratio between switching current and switching voltage. Because the devices show a sharp transition between the LRS and the HRS, it is not difficult to extract *R*. For the switching current (Fig. 6a for the Set and Fig. 7a for the Reset), there exists a scaling behavior following a power-law relation (*I* ∝ *R*^−γ^) in our RS devices. From the least-squares curve fitting (solid lines), we find the exponent γ to be 0.99 ± 0.025 for the Set and 0.97 ± 0.038 for the Reset. The γ value of 0.97 ± 0.038 for the Reset is larger by ~25% than reported values of ~0.7 ± 0.1 in the high resistance regime^21^. However, in considering different ways to define the switching resistance, the γ values appear comparable. On the contrary, the switching voltage seems to have no such scaling effects (Fig. 6b for the Set and Fig. 7b for the Reset). There is no consistent trend in the switching voltage when increasing the switching resistance for individual RS devices.

Figures 6c and 7c show the switching power (*P*) vs *R*, taken at the Reset and Set transitions. The switching power for the Reset (*P*_*Reset*_) and Set (*P*_*Set*_) processes is defined as a product of switching current and switching voltage in each resistance state (See Supplementary Information, Fig. S2). As *R* increases the switching power *P* for both Set and Reset processes decreases with the similar empirical power-law expression: *P* = α*R*^−β^ where α and β are constants. The solid lines represent the fitting curves. The exponent β value is found to be 0.96 ± 0.044 for Set and 1.12 ± 0.078 for Reset. Interestingly, the obtained β values are comparable (See Supplementary Information, Table S1). However, α is found to be 5.04 ± 1.49 for the Set and 0.57 ± 0.13 for the Reset. The much larger α value for the Set process means that approximately 10 times more electrical power is required for the Set process at similar *R* values.

Assuming that one filament having the lowest-resistance plays a dominant role in determining the LRS *I*–*V* characteristics, the metallic ohmic-like LRS transport can be described by the conventional drift current-voltage (*I*_*LRS*_−*V*) model:


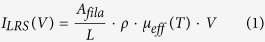


where *A*_*fila*_ and *L* are the effective area and length of the filament respectively. Note again that *A*_*fila*_ is not the area of the pad used. *ρ* and *μ*_*eff*_ represent the charge density and the effective mobility of electrons in the main low-resistance filament. Both *ρ* and *μ*_*eff*_ are parameters peculiar to the materials. As the current increases the effective temperature of the filament also increases. When the temperature becomes high enough to allow the thermo-chemical reaction induced rupture of the filament, the LRS→HRS transition occurs. Because smaller *R* values mean thicker filaments (larger *A*_*fila*_), *P*_*Reset*_ required for disconnecting narrower filaments is higher^36^. As the LRS current increases beyond a critical value, the thermo-chemical reaction starts breaking the thermo-chemically weakest part of the metallic filament. The experimentally observed power-law relation (or universality) between *P*_*Reset*_ and *R* is indicative that as *A*_*fila*_/*L* of a conducting filament increases linearly the required *P*_*Reset*_ increases according to the power law. Though individual RS devices have different material parameters and heat dissipation properties which affect the switching properties^37^,^38^, the experimental observations suggest that the thermal electro-chemical reaction responsible for the rupture of the filament (Reset) is related to the universality described by the power law equation of *P* ∝ *R*^−β^.

**Figure 8:**
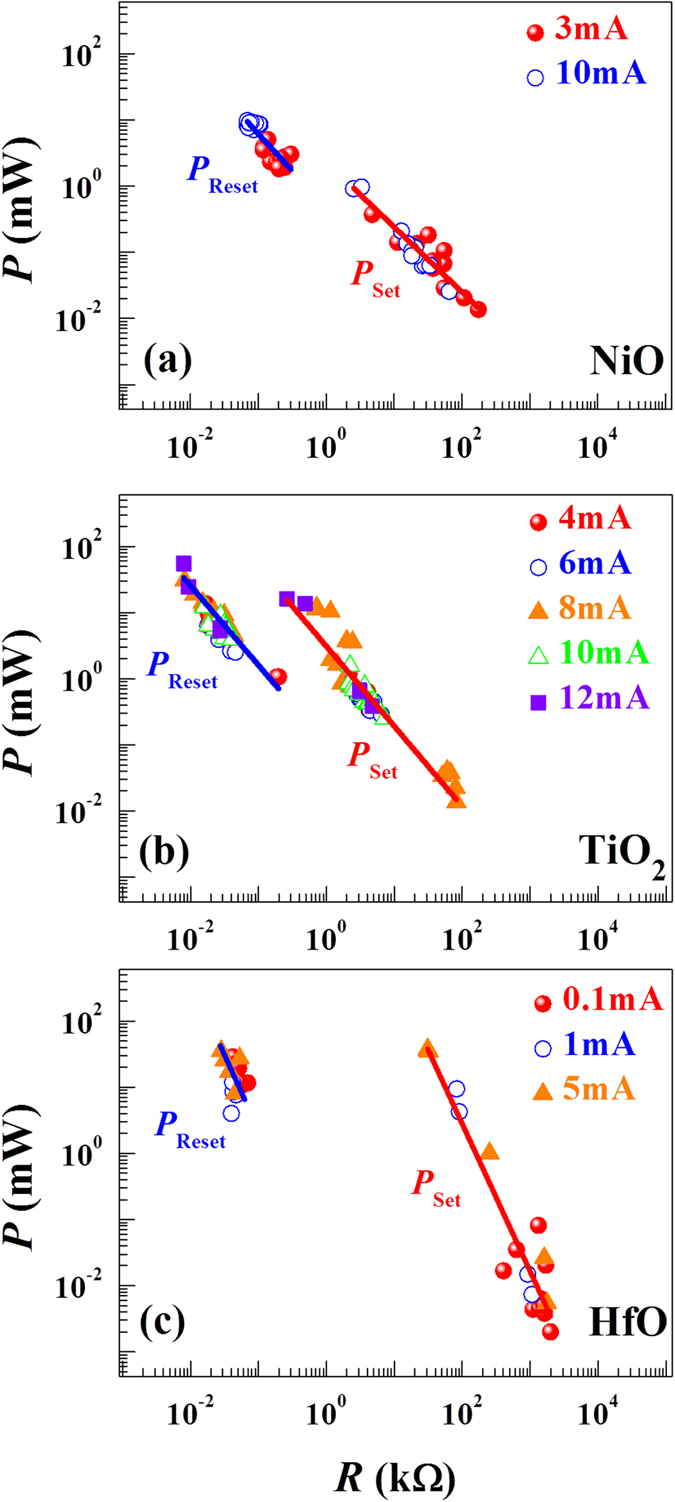
Switching power (*P*) as a function of switching resistance (*R*) for different current compliance values. (**a**) NiO, (**b**) TiO_2_ and (**c**) HfO. The similar power universality is observed regardless of the applied compliance value. The solid lines represent data fitting with a power law relationship.

Figure 8 shows the switching power versus switching resistance data for various current compliance limit values. These NiO-, TiO_2_- and HfO-based RS devices shows the dependence of the LRS current on the pre-set current compliance value. As the compliance value increases, the LRS current also increases proportionately. Because the thickness of the film is fixed, the *I*_*LRS*_ current is determined mainly by the effective area of the filament (*A*_*fila*_). As evident in these results, a power-law relation between *R* and *P*_*Reset*_ is detected validating the switching power universality and corroborating the idea that the effective area *A*_*fila*_ of the filament plays an important role in determining the switching power for the LRS→HRS transition. A similar power universality is also observed for the Set process indicating that the nature of the filamentary conduction channels formed in the previous LRS plays a crucial role in determining the HRS→LRS transition. For the HRS→LRS transition after the forming process, the basic mechanism could be described by a thermo-chemical dielectric breakdown model^39^. This model suggests that the enthalpy of activation for bond breakage and local electric field plays a key role in the breakdown process. These parameters are presumably dependent on structural and electronic properties of the switching oxide medium after the forming process.

In addition, our experimental findings are obtained in the relatively high resistive regime (*R*_*Reset*_ > 10 Ω and *R*_*Set*_ > 10^3^ Ω). Thus, it would be interesting and worthwhile to elucidate whether analogous power universal behaviors in the low resistive region (where different scaling effects in the switching current are observed^21^ and the microscopic nature of the switching medium is expected to differ accordingly) exist.

## Conclusions

In summary, we have fabricated various binary metal oxide-based RS memory devices and investigated their reversible unipolar RS characteristics. We find universality between switching power and resistance. The switching power shows a power-law decrease with increasing switching resistance. For the Reset process (LRS→HRS), this universality can be described in the framework of the conducting filament model, or vice versa, the observed power universality proves the existence of a common behavior in the filament model. For the Set process (HRS→LRS), a similar power-law relation between switching power and resistance is observed, but it is found that larger electrical power is needed, by as much as one order of magnitude at a similar switching resistance. Though the data analysis is based mainly on binary metal oxides, the overall experimental findings in this work can be further extended to other systems such as nitride films which also show unipolar RS whose origin is understood in terms of the same filament model. These experimental findings for the power universality advance the understanding of the filament model for the unipolar RS phenomena and are also useful for device and circuit engineers to perform advanced research on non-volatile RS memory devices.

## Method

The experimental details of the ReRAM device structure and film growth are summarized in Table 1. The two terminal current-voltage measurements (*I*–*V*) were performed using a standard voltage source and current amplifier system (Keithley 4200 system). A bias voltage was applied to the top electrode keeping the bottom electrode to be grounded.

## Additional Information

**How to cite this article**: Kim, J. *et al.* Switching Power Universality in Unipolar Resistive Switching Memories. *Sci. Rep.* **6**, 23930; doi: 10.1038/srep23930 (2016).

## Supplementary Material

Supplementary Information

## Figures and Tables

**Table 1:** Fabrication parameter values of used ReRAM devices and growth method.

Structure	Switching film Thickness	Growth Temperature	Partial pressure or working pressure	Method
Pt/NiO/Pt	300 nm	RT	Ar/O_2_ = 20:4, 17%	DC magnetron sputtering, ref. 40
	350 nm	250 °C	Ar/O_2_ = 27:3, 10%	
	400 nm	RT	Ar/O_2_ = 27:3, 10%	
Pt/TiO_2_/Pt	100 nm	RT	Ar/O_2_ = 2:8, 80%	RF-sputtering
	50 nm	RT	Ar/O_2_ = 2:8, 80%	
Pt/Nb_2_O_5_/Pt	40 nm	200 °C		Pulsed laser deposition, ref. 41
Al/Al_2_O_3_/Al	100 nm	RT		Anodizing technique
Pt/HfO/Ti	10 nm	RT	Ar/O_2_ = 10:1.5, 2 mTorr	Inductively coupled RF-sputtering
Pt/MgO/CuAg/MgO/Ag	50 nm 100 nm	RT	Ar/O_2_ = 10:2, 3 mTorr	RF-sputtering
Pt/CoO/Pt	50 nm	RT	Ar = 15 sccm, 2 mTorr	DC magnetron sputtering, ref. 42
Ti/AlN/Ti	70 nm	RT	Ar/N_2_ = 10:3, 3 mTorr	RF-sputtering
Ti/MnO_2_/Pt	80 nm	650 °C	Ar/O_2_ = 5:5, 50%	RF-magnetron sputtering with Mn target
