# Supplementary material for: Switching Power Universality in Unipolar Resistive Switching Memories
Source: Sci Rep. 2016 Apr 1;6:23930. doi: 10.1038/srep23930 (PMC4817028; doi:10.1038/srep23930)
Supplement: Supplementary Information [file srep23930-s1.pdf]

# Supporting Information

## SWITCHING POWER UNIVERSALITY IN UNIPOLAR RESISTIVE SWITCHING MEMORIES

Jongmin Kim[1], Kyooho Jung[1,2], Yongmin Kim[1,3], Yongcheol Jo[1], Sangeun Cho[1], Hyeonseok Woo[1], Seongwoo Lee[1], A. I. Inamdar[1], Jinpyo Hong[4], Jeon-Kook Lee[5], Hyungsang Kim[1], Hyunsik Im[1]

[1]Division of Physics and Semiconductor Science, Dongguk University, Seoul 100-715, Republic of Korea. [2]R&D Division, Flash Integration Technology Team, SK-Hynix, 2091 Gyeongchung daero Bubal-eub, Icheon-si Gyeonggi-do, Republic of Korea. [3]LED Product & Technology, LED Business, Samsung Electronics Co., Ltd, San #24 Nongseo-dong, Giheung-gu, Yongin-city, Gyeonggi-do, 446-711, Republic of Korea. [4]Department of Physics, Hanyang University, Seoul 133-791, Republic of Korea. [5]Center for Opto-Electronic Materials and Devices, Korea Institute Science & Technology (KIST), Seoul 136-791, Republic of Korea.

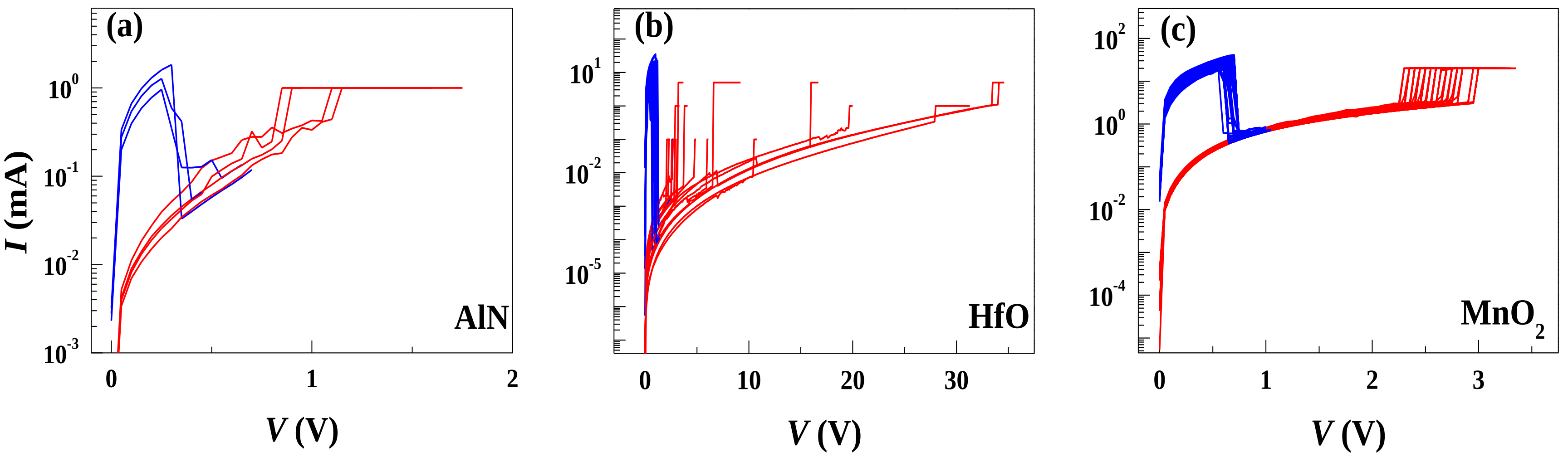


**Fig. S1.** Representative resistive switching current-voltage curves for (a) Ti/AlN/Ti, (b) Pt/HfO/Ti and (c) Ti/$MnO_2$/Pt devices.

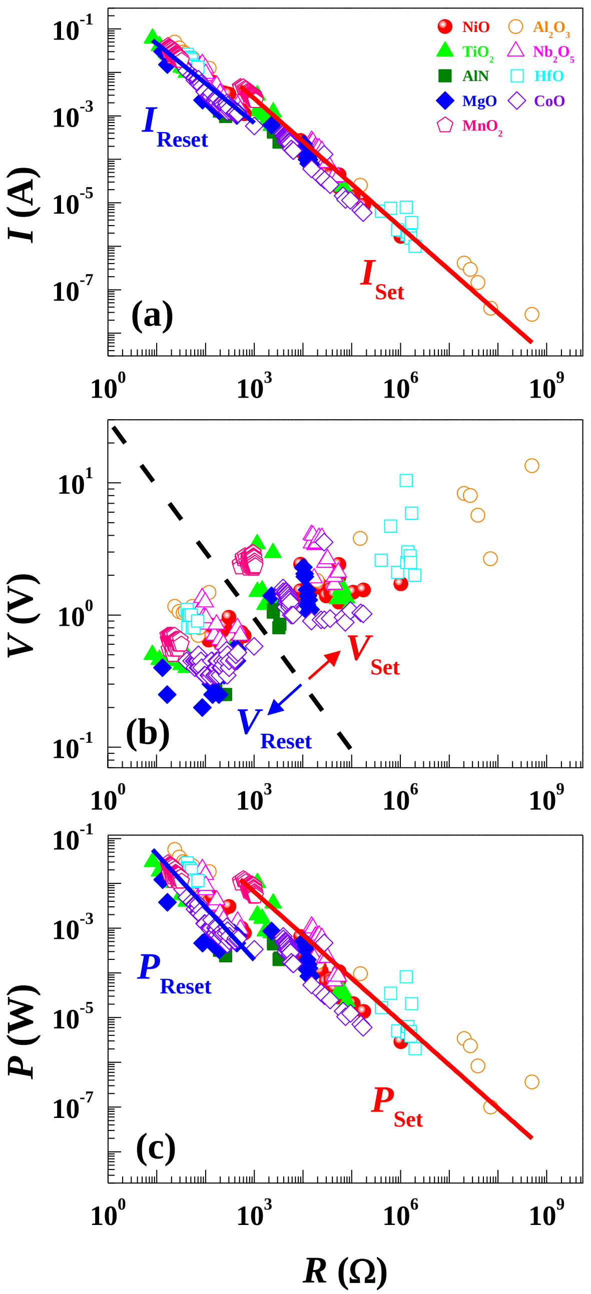


**Fig. S2.** (a) Switching current $I$, (b) switching voltage $V$ and (c) switching power $P$ as a function of switching resistance $R$ for the Set and Reset processes. The solid lines represent the least-squares fitting curves using a $R^{-\beta}$ power-law.

| Material | $\beta_{Set}$ | $\beta_{Reset}$ |
|---|---|---|
| NiO | 0.92±0.219 | 0.93±0.232 |
| $Al_2O_3$ | 0.68±0.429 | 1.49±0.193 |
| $TiO_2$ | 1.20±0.133 | 1.16±0.128 |
| $Nb_2O_5$ | 2.03±0.552 | 1.55±0.509 |
| HfO | 0.93±0.878 | 1.76±0.618 |
| MgO | 1.10±0.224 | 1.10±0.849 |
| CoO | 1.17±0.251 | 1.05±0.074 |
| $MnO_2$ | 0.98±0.076 | 1.35±0.079 |
| Average | 1.15±0.363 | 1.35±0.350 |

**Table. S1.** Extracted exponent β values for individual RS devices. RS devices with a large number of switching cycles are used. The average value of the individually obtained β parameters is comparable to the β value obtained from the full least-squares fitting, validating the power-law relation between switching power and switching resistance.

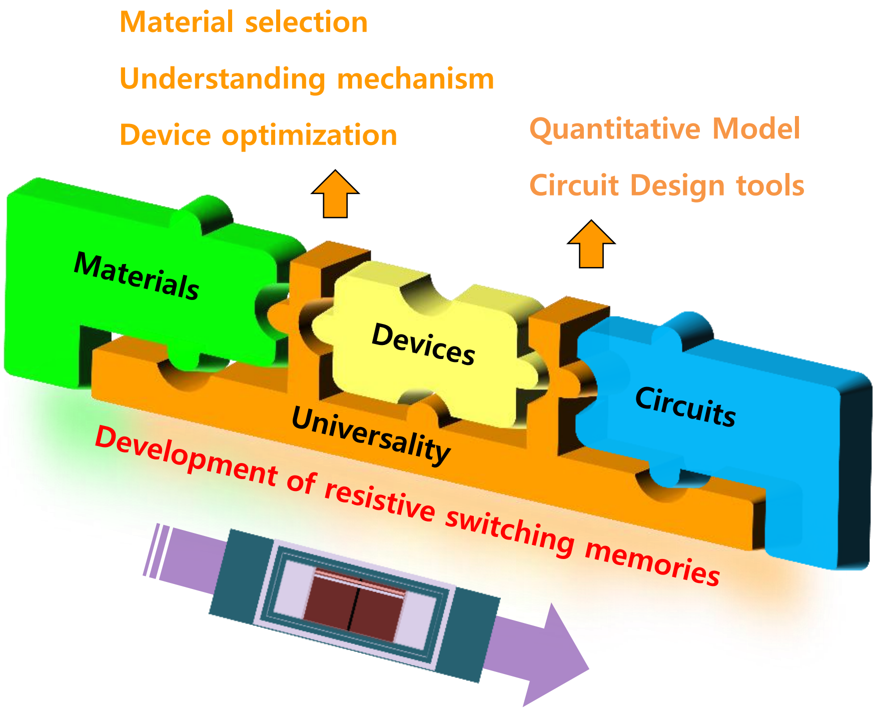


**Fig. S3. Importance of quantitative universality for the development of resistive switching memories.**
The quantitative universality of device parameters in various resistive switching devices can provide a deeper understanding of the correlation between materials, devices and circuits, regardless of different process integration schemes used in the ReRAM industry.
